# Using machine learning to create a decision tree model to predict outcomes of COVID-19 cases in the Philippines

**DOI:** 10.5365/wpsar.2021.12.3.831

**Published:** 2021-09-14

**Authors:** Julius R. Migriño, Ani Regina U. Batangan

**Affiliations:** a San Beda University College of Medicine, Manila, Philippines.; b School of Medicine and Public Health, Ateneo de Manila University, Pasig City, Philippines.

## Abstract

**Objective:**

The aim of this study was to create a decision tree model with machine learning to predict the outcomes of COVID-19 cases from data publicly available in the Philippine Department of Health (DOH) COVID Data Drop.

**Methods:**

The study design was a cross-sectional records review of the DOH COVID Data Drop for 25 August 2020. Resolved cases that had either recovered or died were used as the final data set. Machine learning processes were used to generate, train and validate a decision tree model.

**Results:**

A list of 132 939 resolved COVID-19 cases was used. The notification rates and case fatality rates were higher among males (145.67 per 100 000 and 2.46%, respectively). Most COVID-19 cases were clustered among people of working age, and older cases had higher case fatality rates. The majority of cases were from the National Capital Region (590.20 per 100 000), and the highest case fatality rate (5.83%) was observed in Region VII. The decision tree model prioritized age and history of hospital admission as predictors of mortality. The model had high accuracy (81.42%), sensitivity (81.65%), specificity (81.41%) and area under the curve (0.876) but a poor F-score (16.74%).

**Discussion:**

The model predicted higher case fatality rates among older people. For cases aged > 51 years, a history of hospital admission increased the probability of COVID-19-related death. We recommend that more comprehensive primary COVID-19 data sets be used to create more robust prognostic models.

A novel coronavirus that causes severe respiratory symptoms was first detected in patients in Wuhan City, Hubei Province, China in December 2019. The World Health Organization declared the outbreak of coronavirus disease 2019 (COVID-19) caused by severe acute respiratory syndrome coronavirus 2 (SARS-CoV-2) (*1*) to be a public health emergency of international concern on 30 January 2020, and declared a pandemic on  11 March 2020. As of 22 August 2021, the Philippines’ Department of Health (DOH) had tallied 1 839 635 total cases, 125 900 of which were active cases; there have been 1 681 925 recoveries and 31 810 deaths. (*2*) At that date, the virus had infected more than 209.9 million people and claimed more than 4.4 million lives worldwide. (*3*)

SARS-CoV-2 infection can cause a range of symptoms, from a common cold-like illness presenting with cough, dyspnoea, dysgeusia and fever to severe respiratory symptoms causing shock and multiorgan failure. (*1*) Cases in the Philippines are classified as mild, moderate, severe or critical. (*4*) According to Urwin, Kandola and Graziado (2020), (*5*) a community-friendly prognostic clinical risk prediction score for COVID-19 mortality, severity and complications and triage recommendations can be determined from current signs and symptoms, comorbidities, medical history and demographics. In that study, the demographic factors of risk included age, sex, country and partial postcode.

Predictive modelling could greatly help low- and middle-income countries such as the Philippines to address the COVID-19 pandemic by increasing the accuracy of diagnosis and prognosis of patients. (*6*, *7*) This may help in determining the outcomes and factors indicative of outcomes for patients with COVID-19. Predictive modelling may also aid policy-makers in determining which strategies are more effective, so that allocation of limited resources can be targeted to possible target populations more efficiently and cost-effectively, especially during a pandemic. (*6*) Predictive modelling may also help to inform patients about the possible course of their illness and help both patients and health-care workers to draw up diagnostic and therapeutic plans. (*8*)

Machine learning and artificial intelligence have been used to automate the detection of patterns in large data sets, especially in dealing with the massive amounts of data generated during a global event such as the current pandemic. Decision trees, a specific type of machine learning, are based on covariates to create a model for predicting outcomes. (*9*) Currently, artificial intelligence, including decision tree modelling, is being used in the COVID-19 pandemic for early detection and diagnosis, monitoring treatment, tracing contacts, developing drugs and vaccines, predicting cases and fatalities and even identifying the most vulnerable groups. (*10*-*13*) Machine learning has been used to identify demographic and clinical predictors of disease progression, which include age, sex, body temperature, associated signs and symptoms, minimum oxygen saturation, computed tomography scan features, C-reactive protein and lactic dehydrogenase levels and lymphocyte counts. (*12*, *14*)

In the Philippines, studies on COVID-19 modelling have been limited to compartmental models, such as “susceptible–infectious–recovered/removed” or “susceptible–exposed–infectious–recovered/removed” models, (*15*, *16*) usually for tracking epidemiological trajectories. Other types of models being used in the Philippines include regression analysis models to estimate case fatality rates (*17*) and to determine socioeconomic indicators of the number of cases. (*18*)

The aim of this study was to create a decision tree model with machine learning to predict outcomes (i.e. recovery or death) of COVID-19 cases based on publicly available data from the DOH COVID Data Drop.

## Methods

We used the publicly available DOH COVID Data Drop database for 25 August 2020. (*19*) This database is extracted from the COVID-19 information system by the DOH Epidemiology Bureau and is updated daily. The data are obtained from paper-based case investigation forms from all the regional epidemiology surveillance units in the country. The raw data set comprised 197 164 cases, which represented all reported COVID-19 cases with at least one positive reverse transcription-polymerase chain reaction test of a respiratory swab. The raw data set was filtered to include only resolved cases (i.e. cases with an entry under the attribute *RemovalType*), as unlabelled cases are still active. *RemovalType* was defined as the outcome for the patient and was coded as either “RECOVERED” or “DIED.” Descriptive statistics, i.e. means, standard deviations, case fatality rates (CFR), *t*-tests (for continuous variables) and Pearson’s *χ*^2^ tests (for nominal variables) were generated with StataCorp 2013 (Stata Statistical Software, Release 13; College Station, TX).

CFR (%) = 

We conducted an exploratory analysis to screen cases and attributes in the raw data set. The attribute *AgeGroup* was recoded to reclassify *Age* (age of patient, in years) into nine ranges according to the classification of the United States Centers for Disease and Control and Prevention. (*20*) *Pregnanttab* was defined as a binary variable representing whether a patient was pregnant at any time during COVID-19 infection, with male cases coded as missing values. Missing values for *CityMunRes* (patient’s city of residence) and *ProvRes* (patient’s province of residence) were recoded as “Repatriate” for all cases with *RegionRes* (patient’s region of residence) = “Repatriate.” The data set was then filtered to select only cases with no other missing values to generate the final data set. Details of the data pre-processing can be found in **Supplementary Information A**.

Attribute selection, random undersampling, hyperparameter optimizations, model generation, cross-validation and performance calculations were done in RapidMiner Studio 9.7.002 (rev. db1bb6, platform: WIN64) (see **Supplementary Information C**). The attribute *RemovalType* was labelled as the outcome in the data set. Attributes were selected with feature weights operators (*weightbyGiniIndex*, *weightbyInformationGain*, *weightbyInformationGainRatio*) to determine those appropriate for model generation. The subprocess *optimizeParameters(Grid)* was used to perform grid optimization of the hyperparameters for the decision tree operator *decisionTree* and the threshold operator *createThreshold*. The subprocess ran a fivefold cross-validation operator to train and validate the data set with the decision tree model and the optimized *decisionTree* and *createThreshold* hyperparameters generated for each fold. Random undersampling was done only on the training data set for each fold in the cross-validation operator, with the sample operator to (i) select all cases with *RemovalType* = DIED and (ii) randomly select cases with *RemovalType* = RECOVERED using stratified sampling to achieve a 1:1 RECOVERED:DIED ratio. Stratified sampling generated two subsets from the modelling data set that ensured similar *RemovalType* case distribution (i.e. RECOVERED and DIED) between the two subsets by simple random sampling. All cases in the testing data set were used to validate the model for each fold in the cross-validation.

The decision tree model generated by the cross-validation training data set was also extracted. Performance metrics such as area under the curve (AUC), accuracy, F-score, sensitivity and specificity were extracted from the cross-validation with the positive class set as *RemovalType* = DIED. Similar cross-validation operators were used to train and validate a naïve Bayes model for comparison. Details of the model generation can be found in **Supplementary Information B**. The study adhered to the TRIPOD checklist for prediction model development. (*21*)

### Ethics statement

The study was reviewed and approved on 19 August 2020 by the San Beda University Research Ethics Board under the study protocol code SBU-REB 2020–017. The study adhered to the TRIPOD checklist for prediction model development.

## Results

### Description of cases

The final data set was a list of 132 939 resolved COVID-19 cases (98.16% of all resolved cases and 67.43% of total reported cases from the raw data set). Of the reported cases, 97.7% recovered and 2.3% died. There were more COVID-19 cases among males than females (145.67 per 100 000 vs 118.10 per  100 000; *P* < 0.001). CFRs were also higher among males than females (2.46% vs 1.97%; *P* < 0.001). The most resolved cases were among people aged 18–29 years. Cases aged (*3*)85 years had the highest CFR (22.57%), followed by those aged 75–84 years (17.99%) and 65–74 years (12.01%). The age group 18–29 years had the lowest CFR, at 0.27% (**Table 1**). Disaggregation of male and female cases showed similar patterns of cases by age group (**Table 2**).

**Table 1:** Demographic characteristics of resolved cases (recovered or died) from the Philippines COVID Data Drop from 25 August 2020

-	Recovered	Died	CFR (%)	*P* < 0.001
Sex, *n* = 135 434	-	-	-	-
Male	73 919	1863	2.46	-
Female	58 477	1175	1.97	-
Age, *n* = 133 097	-	-	-	< 0.001
Mean age (years)	38.05 (± 15.93)	61.33 (± 16.73)	-	-
Age group (years), *n* = 133 097	-	-	-	< 0.001
0–4	1830	32	1.72	-
5–17	5563	26	0.47	-
18–29	37 080	100	0.27	-
30–39	32 632	147	0.45	-
40–49	22 315	294	1.30	-
50–64	21 907	995	4.34	-
65–74	6148	839	12.01	-
75–84	2092	459	17.99	-
^3^ 85	494	144	22.57	-
Region, *n* = 131 614	-	-	-	< 0.001
BARMM	455	11	2.36	-
CAR	370	8	2.12	-
CARAGA	297	4	1.33	-
NCR	74 572	1430	1.88	-
Repatriate	6586	15	0.23	-
Region I: Ilocos Region	609	26	4.09	-
Region II: Cagayan Valley	483	3	0.62	-
Region III: Central Luzon	3850	81	2.06	-
Region IV-A: CALABARZON	17 201	253	1.45	-
Region IV-B: MIMAROPA	396	7	1.74	-
Region V: Bicol Region	773	22	2.77	-
Region VI: Western Visayas	1865	48	2.51	-
Region VII: Central Visayas	16 256	1006	5.83	-
Region VIII: Eastern Visayas	1302	8	0.61	-
Region IX: Zamboanga Peninsula	889	37	4.00	-
Region X: Northern Mindanao	766	16	2.05	-
Region XI: Davao Region	1480	56	3.65	-
Region XII: SOCCSKSARGEN	429	4	0.92	-

BARMM: Bangsamoro Autonomous Region in Muslim Mindanao; CAR: Cordillera Administrative Region; CARAGA: Caraga Administrative Region; NCR: National Capital Region; CALABARZON: Batangas, Cavite, Laguna, Quezon, Rizal and Lucena; MIMAROPA: Mindoro, Marinduque, Romblon and Palawan; SOCCSKSARGEN: South Cotabato, Cotabato, Sultan Kudarat, Sarangani and General Santos.

**Table 2:** Resolved cases (recovered or died) from the Philippines COVID Data Drop from 25 August 2020 by age group and sex (*n* = 133 097)

Age group (years)	Males (*n* = 74 395)			Females (*n* = 58 702)			CFR ratio^a^
	Recovered	Died	CFR (%)	Recovered	Died	CFR (%)	
0–4	978	18	1.81	852	14	1.62	1.12
5–17	2848	12	0.42	2715	14	0.51	0.82
18–29	19 967	61	0.30	17 113	39	0.23	1.30
30–39	18 995	97	0.51	13 637	50	0.37	1.38
40–49	13 397	191	1.41	8918	103	1.14	1.24
50–64	12 001	647	5.12	9906	348	3.39	1.51
65–74	3157	506	13.81	2991	333	10.02	1.38
75–84	1012	266	20.81	1080	193	15.16	1.37
^3^ 85	178	64	26.45	316	80	20.20	1.31

^a^ CFR ratio is computed as *CFR males/CFR females*

The highest notification rates of COVID-19 cases were from the National Capital Region, followed by Regions VII and IV-A (590.20, 285.70, 121.08 per 100 000, respectively). The highest CFR was observed in Region VII (5.83%), followed by Regions I (4.09%) and IX (4.00%). The lowest CFR was observed among repatriates (0.23%), followed by Regions VIII (0.61%) and II (0.62%). Although the National Capital Region and Region IV-A had the most cases, they had low case fatality rates (1.88% and 1.45%, respectively) (**Table 1**).

### Outcomes from machine learning models

**Figure 1:**
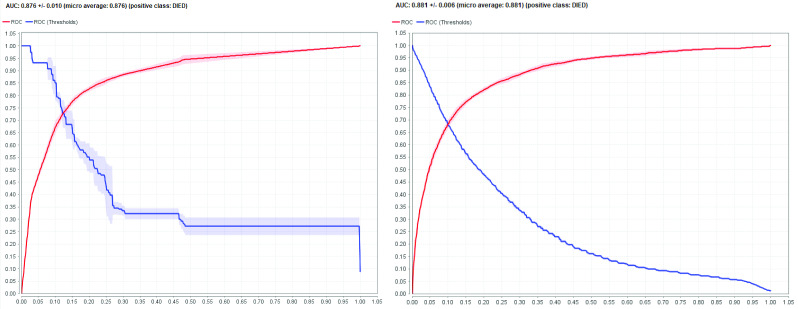
Receiver operating characteristic (ROC) curves for the two machine learning models: decision tree and naïve Bayes^a^

Of the three feature weighting operators, only the attributes Age and Admitted were included in the final model. The decision tree model was trained and cross-validated with the following optimized hyperparameters: criterion = information_gain, maximal_depth = 8, minimal_gain = 0.0, minimal_leaf_size = 10, minimal_size_for_split = 100. The comparator naïve Bayes model used the same optimized hyperparameters in model training and cross-validation and had a higher AUC (0.881 ± 0.006), accuracy (81.68% ± 0.05%), F-score (16.75% ± 0.33%) and specificity (81.71% ± 0.52%) and a better receiver operating characteristic (ROC) curve. The decision tree model had greater sensitivity (81.65% ± 1.64%) (**Table 3**; **Fig. 1**).

[**insert** Figure 1]

**Table 3:** Performance metrics for the two machine learning models: decision tree and naïve Bayes using the modelling data set and optimized hyperparameters

Model	AUC		Accuracy		F-score		Sensitivity		Specificity
Decision tree	0.876	± 0.010	81.42%	± 1.01%	16.74%	± 0.55%	81.65%^a^	± 1.64%	81.41%
Naïve Bayes	0.881^a^	± 0.006	81.68%^a^	± 0.05%	16.75%^a^	± 0.33%	80.63%	± 1.17%	81.71%^a^

^a^ Highest values for each metric across all models

**Figure 2:**
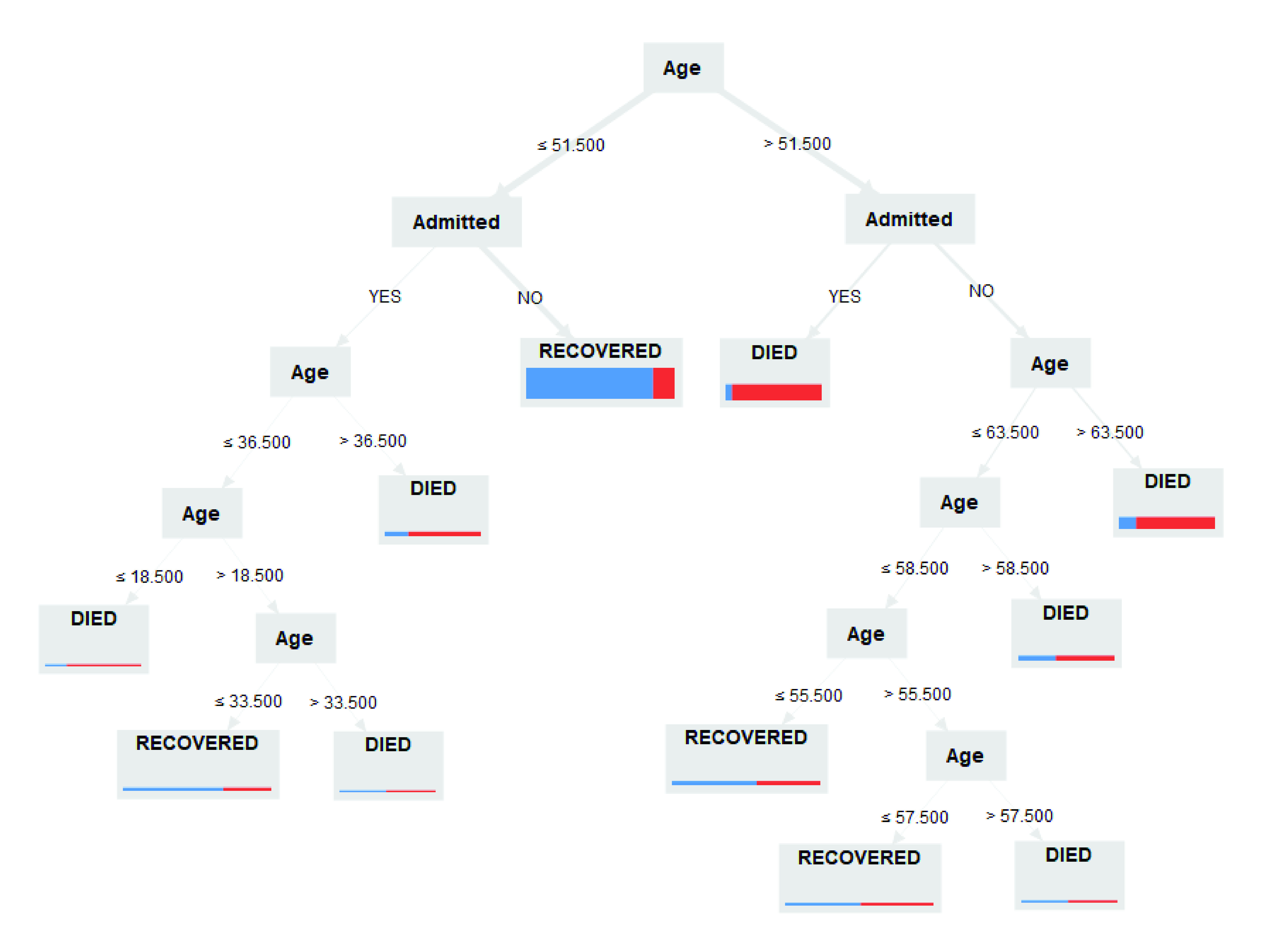
Decision tree for predicted outcomes of resolved cases (recovered or died) from the Philippines COVID Data Drop from 25 August 2020^a^

The decision tree had seven levels, with each node splitting into two branches or leaves (**Fig. 2**; **Supplementary Information B5** provides other details of the decision tree, including the actual number of cases and outcomes per leaf). The primary (root) node was Age, with the split criterion being a cut-off of 51.5 years, based on the average of the split criterion of the values Age = 51 and Age = 52. The attribute Admitted split the lower branches further, with further splits according to Age. The majority of all cases in the model (53.54%) were < 51.5 years and had no history of hospital admission, and most recovered (85.46%). Similarly, the majority of cases aged 51.5–57.5 years with no history of hospital admission recovered (55.14%). There were increasing proportions of deaths with increasing age, with the highest death rates among those > 63.5 years (81.98%). A high proportion (93.33%) of people aged > 51.5 years with a history of hospital admission died.

[**insert** Figure 2]

## Discussion

Using a decision tree model, we generated a simple seven-level, multinodal decision tree to predict COVID-19-related outcomes of reported cases in the Philippines on the basis of the attributes of age and hospital admission. Tree-based methods in the classification and regression tree paradigm are increasingly used and “have become one of the most flexible, intuitive, and powerful data analytic tools for exploring complex data structures.” (*22*) Modern programming software and applications have enabled the use of machine learning algorithms such as decision trees to process large sets of data (*23*, *24*) and are widely used in health care, including as prediction models. (*9*, *25*-*27*) Decision trees are also easy to understand and interpret, (*13*, *28*) which is useful for both health workers and policy-makers, and are flexible enough to handle non-parametric class densities such as data from COVID-19 databases. (*29*)

Our decision tree model indicated age as the main predictor of clinical outcomes for COVID-19. In both the descriptive analysis and the decision tree model, younger cases had higher recovery rates, while older age groups had noticeably higher mortality rates, regardless of hospital admission status. These findings are consistent with the current international literature (*20*, *30*-*32*) as well as locally reported data. (*2*) The age cut-off of 51.5 years determined in the decision tree model was, however, lower than the current age cut-off used in most Philippine medical (*4*) or policy guidelines, suggesting that age cut-offs (both lower and upper bounds) for guidelines should be re-evaluated continually. The youngest age group in this study (0–17 years old) had higher case fatality rates than the baseline (18–29 years old), which was consistent even for cases < 19 years of age with a history of hospital admission, who had a high death rate. This finding is inconsistent with the available literature but may be due to the relative paucity of confirmed cases and studies in younger COVID-19 cases. Alternatively, it may be due to the fact that age-differentiated studies have been conducted with data from developed countries, such as China, England and Wales, France, the Republic of Korea and Spain, (*30*, *33*) and may not be comparable to the situation in the Philippines.

A history of hospital admission was another strong predictor of mortality from COVID-19, especially in cases > 51 years of age. Rationally, this is to be expected, as current national guidelines for hospitalization of COVID-19 patients are for those risk-stratified as moderate, severe or critical. (*4*) Our study affirms the use of the current guidelines in the Philippine setting.

Although sex was not a predictor in the decision tree model, males had a statistically significantly higher CFR fatality rate than females in the descriptive analysis. This difference in the adult population is consistent with that found in an international study by Bhopal and Bhopal using pooled data from multiple countries, with higher male:female CFRs for age groups (*3*)40 years (range: 1.65–2.6). (*33*) Sex differentials in COVID-19 mortality have been extensively documented in multiple studies, (*32*-*34*) and our study confirmed these findings in the Philippine setting. Proposed mechanisms for a sex differential include the fact that males generally have more pre-existing comorbidities like hypertension, cardiovascular disease and chronic obstructive pulmonary disease; poorer health behaviours (e.g. smoking and drinking alcoholic beverages); and even biological differences, such as specific receptor regulation, chromosomal variation and differences in interferon and hormone levels. (*34*)

Despite differences in responses to COVID-19, notification rates and CFRs in different geopolitical classifications (i.e. city/municipality, province or region) were not seen in the model. **Supplementary Information B2** provides details of the feature weights for city/municipality, province and region as attributes. This result suggests that guidelines can be national, albeit with a subgroup-targeted approach, for clinical and public health management, primarily based on disease interaction with age and sex, (*1*, *33*-*35*) and specifically focusing on the increased risk of males and older age groups for death from COVID-19. (*33*, *35*)

Our study has two types of limitations: the quality of the data set and model limitations. The quality of the data set was compromised mainly by availability and data points from the raw data set. As the DOH COVID Data Drop is publicly available, measures are in place to protect the privacy and confidentiality of sensitive patient information. Thus, some useful information potentially associated with COVID-19 mortality, such as presence of comorbidities, smoking history, travel history, exposure history to a confirmed COVID-19 patient, clinical signs and symptoms of disease processes and poor laboratory findings, (*1*, *11*) were not included in the initial data set and were therefore not included in our model. Furthermore, the DOH COVID Data Drop reported multiple instances of duplicate and missing entries (*19*) on different dates. Additionally, a potentially relevant attribute, HealthStatus (defined as “Asymptomatic,” “Mild,” “Severe,” “Critical,” “Recovered” or “Died”), was not included in the model, as its values change constantly, with no time stamp. We suggest that future studies use primary COVID-19 data sets that include these parameters for more robust prognosis modelling, such as case investigation forms from the DOH Epidemiology Bureau or from hospital records (e.g. PhilHealth claim form 4).

The model generally had high-performance metrics: AUC, accuracy, sensitivity and specificity were reasonably high; however, its calculated F-score was low due to poor model precision. In classification trees for disease diagnostics and prognostics, high sensitivity is preferred to accuracy, (*25*) especially in inherently imbalanced data sets such as COVID-19 prognosis databases. We tried to control for this imbalance by undersampling, which is more resistant to overestimation of predictive accuracy than oversampling techniques. (*36*, *37*) Other model limitations include the inherent propensity of decision trees for “over-fitting,” which often occurs in highly complex models for relatively simple data, which often capture too much noise from the data set. (*25*, *38*) We tried to reduce over-fitting with the following strategies: (i) conducting exploratory data set analysis to remove ambiguous, highly correlated or incompletely filled attributes; (ii) enabling pre-pruning and pruning during model training to limit the complexity of the model; and (iii) running decision tree grid optimization to determine which hyperparameter values would net the highest AUC. Another limitation of the model is sampling bias, in which active cases are excluded from the analysis. This limitation is related to the cross-sectional design of the study and the continuing evolution of COVID-19 in the Philippines. Comparable performance metrics in our study indicate that other classification models, such as naïve Bayes, random forest or deep learning, might be considered for future prognostic models.

In conclusion, our study showed that increasing age and history of hospital admission are important predictors of COVID-19 prognosis, consistent with the current literature. We were able to generate a sensitive, specific decision tree model with a high AUC and with Age and Admission as the main predictors of COVID-19 prognosis using a publicly available data set. We recommend adaptation of our model with more comprehensive primary COVID-19 data sets to create robust COVID-19 prognostic models that could contribute to a review of clinical and public health guidelines.
